# Sphincter-preserving surgery for recurrent pelvic malignancy using a hybrid procedure of open laparotomy and transanal endoscopic approach

**DOI:** 10.1186/s40792-018-0439-y

**Published:** 2018-04-12

**Authors:** Seiji Ishiguro, Shunichiro Komatsu, Kenichi Komaya, Takuya Saito, Takashi Arikawa, Kenichiro Kaneko, Tsuyoshi Sano

**Affiliations:** Division of Gastroenterological Surgery, Department of Surgery, Aichi Medical University, 1-1 Yazakokarimata, Nagakute, Aichi 480-1195 Japan

**Keywords:** Transanal endoscopic approach, Neoplasm recurrence, Local/SU, Ovarian neoplasms

## Abstract

**Background:**

Surgery for the treatment of recurrent pelvic malignancy is challenging. Sphincter-preserving surgery (SPS) has been applied in limited cases. Transanal endoscopic approach (TEA) has been used for primary rectal cancer, predominantly for hybrid transabdominal-transanal total mesorectal excision. Here, we describe the use of TEA as a hybrid approach in a case of recurrent ovarian cancer.

**Case presentation:**

A 59-year-old woman had recurrence of serous ovarian adenocarcinoma in the vaginal stump, near the site of anastomosis from a rectal resection 18 months previously. We used a hybrid approach comprising conventional open laparotomy and TEA to accomplish sphincter preservation. In addition to sphincter preservation, TEA allowed for the creation of a “terminal” space, which was made by anterior dissection between the rectum and the vagina. We employed TEA to create an opening in the scar tissue along the sacrum, which was used as a “guide” for pelvic dissection to prevent nerve injury. After exteriorization of the tumor, bowel continuity was achieved by hand-sewn coloanal anastomosis with a protective diverting ileostomy. Pathological examination revealed no involvement of the surgical margins. The diverting ileostomy was taken down 8 months postoperatively.

**Conclusion:**

A hybrid approach comprising conventional open laparotomy and TEA allowed for safe and secure SPS and complete excision of a recurrent pelvic malignancy. This hybrid surgical approach expands the use of SPS in highly selected cases.

## Background

Surgery for recurrent pelvic malignancy is hampered by the distortion of tissue planes and associated scarring. Most surgeons prefer a wide resection margin and sacrifice the possibility of restoring intestinal continuity, leading to a high rate of abdominoperineal resection (APR) or pelvic exenteration, occasionally with the resection of bony structures [1–3]. Sphincter-preserving surgery (SPS) has been applied in limited cases for recurrent pelvic tumor after anterior resection of the rectum [4]. However, new surgical approaches to maintain the balance between an R0 resection and the preservation of continence are warranted.

The use of transanal endoscopic approach (TEA) has recently increased worldwide. This technique utilizes flexible and disposable transanal endoscopic platforms and accommodates ordinary laparoscopic instruments [5]. A hybrid technique, transabdominal-transanal total mesorectal excision (TaTME), usually performed laparoscopically, has dramatically advanced the treatment of primary rectal malignancy [6, 7]. TaTME procedures begin with dissection of the deepest area, which offers the obvious advantage that the most difficult procedure is completed first, with precise distal margins secured under laparoscopic guidance.

We considered the combination of conventional open laparotomy together with TEA as SPS as a hybrid procedure for recurrent pelvic malignancy. Our idea was to initially accomplish deep dissection of loose connective tissue and then to further advance into the scar tissue under endoscopic guidance. Here, we applied this hybrid approach to a patient with recurrent ovarian malignancy after previous rectal surgery and obtained successful preservation of the sphincter.

## Case presentation

The patient was a 59-year-old woman who had been treated 18 months previously for serous adenocarcinoma of the ovary. Treatment at that time consisted of total hysterectomy, bilateral salpingo-oophorectomy, omentectomy, pelvic lymphadenectomy, para-aortic lymphadenectomy, and low anterior resection for direct invasion of the rectum. An anastomotic leak between the colon and rectum occurred after surgery and a diverting ileostomy was created. After adjuvant chemotherapy, the stoma was closed 8 months postoperatively. At 18 months after surgery, recurrence of the serous adenocarcinoma at the vaginal stump was detected on gynecologic examination. Radiological examination revealed no evidence of recurrence at any other site. Preoperative colonoscopy findings showed no evidence of the tumor at the prior anastomosis, which was located 5 cm from the anal verge. The patient strongly desired SPS and gave consent for treatment by this new method after a full explanation was provided.

**Fig. 1:**
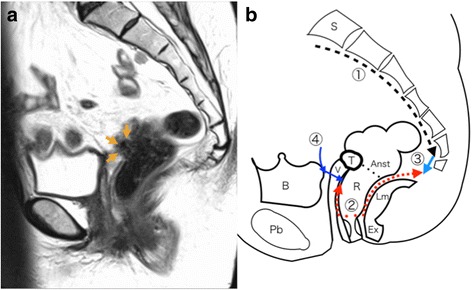
**a** Magnetic resonance imaging (MRI) T2-weighted images. Orange arrows indicate the recurrent tumor in the vaginal stump. **b** Schema of the MRI. Arrow 1, dissection along the anterior sacrum; dotted red arrow 2, dissection by transanal minimally invasive surgery (TEA); light blue arrow 3, subsequent dissection to create a communication between the abdominal dissection and the dissection created by TEA; dark blue arrow 4, vaginal transection in the final phase of surgery. T tumor, S sacrum, B bladder, Pb pubis, V vagina, R rectum, Ex external sphincter muscle, Lm levator muscle, Anst anastomosis from the prior surgery

Conventional laparotomy and adhesiolysis were conducted in the lithotomy position with the legs lowered. The tumor was located around the bladder, vaginal stump, and the prior rectal anastomosis, but no invasion of the bladder was observed. Dissection between the bladder and proximal vagina was performed without difficulty, and the ureters and superior vesical arteries were preserved. The perirectal tissues, particularly around the prior anastomosis, were dense and offered no dissection plane to secure the pelvic plexus. Dissection along the anterior surface of the sacrum was advanced as much as possible using electrocautery toward the deepest part of the pelvis, from which horizontal advancement was limited (Fig. 1, arrow 1).

**Fig. 2:**
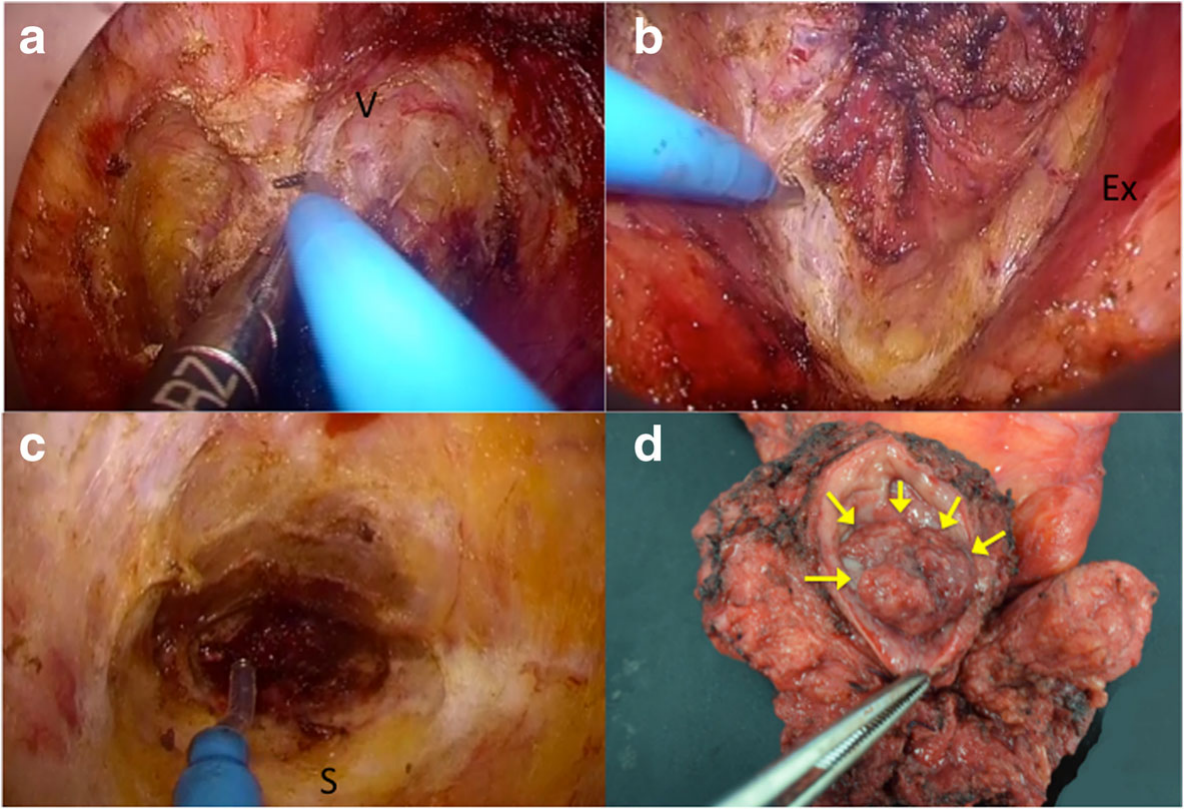
Intraoperative views under the transanal endoscopic approach (TEA). **a** Dissection between the vagina and the rectum. V vagina. **b** Dissection of the external sphincter muscle. Ex external sphincter muscle. **c** Dissection of the scar tissue on the sacrum. S sacrum. **d** The recurrent tumor is located in the vaginal stump, indicated by yellow arrows

The patient was subsequently placed in the lithotomy position with the legs elevated. A full-thickness circumlinear incision of the rectal wall was created inside the anal canal 3 cm from the anal verge, and the proximal stump was closed with a purse-string suture. After irrigation with physiological saline, a multiple access port (GelPOINT Path®, Applied Medical, Inc., Rancho Santa Margarita, CA, USA) was placed into the anus. An AirSeal System® (Conmed, Utica, NY, USA) was used to establish pneumoperitoneum with CO_2_ insufflation and smoke evacuation. An anterior dissection was performed from the anterior rectal wall to the distal two thirds of the vagina using an ordinary flexible laparoscope and a laparoscopic grasper. Care was taken to ensure that the dissection did not advance as far upwards as the tumor. This procedure resulted in a space behind the vagina we termed the “terminal” space, as its creation marks the final goal and end of the combined procedure (Fig. 2a). A dorsolateral dissection was made between the internal and external sphincter muscles and advanced as far as possible through the loose connective tissue between the sphincters (Fig. 2b). The separation unavoidably included scar tissue from the prior surgery on the levator muscle and sacrum. We secured hemostasis under endoscopic guidance (Fig. 2c). The posterior dissection was advanced into the scar tissue over the tip of the coccyx (Fig. 1, arrow 2). The communication to the abdominal dissection could not be completed using the TEA procedure. The lateral dissection was performed to preserve the sacral nerve.

Returning to the abdomen, electrocautery was used to advance farther along the sacral parietal fascia to finally connect to the TEA dissection (Fig. 1, arrow 3), allowing for the creation of a “guidance port” for further dissection. An index finger was inserted into the connection to identify the plane between the rectal fascia and pelvic plexus. After completing the posterior dissection, the anterior and then posterior vaginal wall were sectioned with sufficient margin from the tumor to complete the terminal space behind the vagina made using TEA (Fig. 1, arrow 4). Scar tissue in the deepest part of the pelvis was dissected and the specimen was extracted. Bowel continuity was achieved by hand-sewn coloanal anastomosis, protected by a temporary loop ileostomy. Operation time was 301 min and total blood loss was 441 mL. Pathological examination revealed no involvement of the surgical margins (Fig. 2d). The postoperative course was uneventful and the patient was discharged on postoperative day 18. The diverting ileostomy was taken down 8 months later after adjuvant chemotherapy and following confirmation that there was no evidence of recurrence. After stoma reversal, the patient had frequent bowel passage, which is gradually improving with time, and has not noticed fecal soiling or mucus from the anus.

## Conclusions

This case report demonstrates the first attempt to utilize a hybrid approach comprising conventional open laparotomy and TEA for recurrent pelvic malignancy to achieve sphincter preservation. By starting transanally using the so-called bottoms-up approach using TEA, sphincter preservation was accomplished without any difficulty. Further, TEA allowed for a more advanced horizontal dissection from the perineum to the deepest part of the pelvis under laparoscopic guidance than is possible with conventional APR or intersphincteric resection under direct vision, resulting in safer and more secure surgery. TEA therefore has the potential to expand the indications of SPS to recurrent pelvic malignancy.

TEA has a substantial advantage in pelvic disease recurrence. Regardless of the site of recurrence, adhesions to the sacrum from the initial rectal mobilization are frequently hard and dense even in the absence of a tumor. Additionally, dissection of the scar along the surface of the parietal fascia on the sacrum permits access to the pelvic floor, but further advancement is frequently difficult because the dissection route changes from vertical to horizontal, and direct visualization is limited. In general, diathermy of the scar tissue generates much smoke, which contributes to the poor visibility. The AIR Seal iFS® maintains a clear field with sufficient CO_2_ insufflation and better smoke evacuation, allowing for separation of the scar tissue with secure hemostasis under laparoscopic guidance. In this case, although complete connection to the abdominal dissection could not be created by TEA alone, a subsequent additional dissection from the abdomen provided an opening to the pelvic field and left the levator muscles intact.

The guidance port created using the hybrid procedure is important for allowing sufficient dissection. Distortion from the initial surgery accompanied by dense scar tissue does not usually allow for the creation of an appropriate surgical plane. We predict that this secure and reliable guidance port in the pelvis will be confirmed to play an important role in the safe surgical resection of recurrent pelvic tumors. Insertion of a finger into the abdominal opening allows the dissection plane to be identified. Preservation of the urinary system requires an intact pelvic plexus, uninvolved bladder trigone, and sufficient ureter length. This posterior finger-guided dissection is helpful for identifying the pelvic plexus. The anterior dissection between the vagina and rectum is not difficult under TEA because the dissection plane remains unchanged following the prior surgery and facilitates construction of the terminal space, which is the final goal of the entire pelvic resection. In the present case, the extensive dissection from the posterior guidance port was advanced both laterally and anteriorly, leaving the specimen finally found connected to the tissue around the vagina. Although the region around the vagina can be the site of massive bleeding, the rapid procedure toward the terminal space made it possible to minimize blood loss. We therefore consider it important to emphasize that the creation of both this terminal space and guidance port are essential components of TEA for recurrent pelvic surgery.

The application of TEA to recurrent pelvic tumors should be considered carefully. Currently, TEA for redo pelvic surgery has been described in a small case series necessitated by problems such as anastomotic stenosis, persistent leakage, or severe pouchitis, but in which no recurrent tumors were identified [8]; however, TEA for recurrent pelvic malignancy has not been reported. Resection should be limited without sacrificing the oncologic principle of R0 resection, which may be achieved with extensive and wide resection. With our present limited resection, preoperative magnetic resonance imaging or computed tomography is required to achieve cancer-free margins. It is clear that posterior recurrence, which necessitates resection of the sacrum, is not a candidate for TEA or a hybrid procedure [9]. Our case was of ovarian malignancy, and not rectal adenocarcinoma. Moreover, it is important to keep in mind that different surgical indications are possible depending on the histology. Future studies should evaluate this method for its safety and efficacy, such as with regard to anal function, local recurrence rate, and survival in accumulated cases.

TEA has a substantial advantage not only in primary pelvic surgery but also in redo pelvic surgery. A hybrid approach comprising conventional open laparotomy and TEA in highly selected cases with recurrent pelvic tumors may allow more patients to achieve successful sphincter preservation.

## Abbreviations

APR: Abdominoperineal resection
MRI: Magnetic resonance imaging
SPS: Sphincter-preserving surgery
TaTME: Transabdominal-transanal total mesorectal excision
TEA: Transanal endoscopic approach
